# Therapeutic Effects of the Bcl-2 Inhibitor on Bleomycin-induced Pulmonary Fibrosis in Mice

**DOI:** 10.3389/fmolb.2021.645846

**Published:** 2021-10-07

**Authors:** Yicheng He, Fei Li, Chao Zhang, Xinwei Geng, Madiha Zahra Syeda, Xufei Du, Zhehua Shao, Wen Hua, Wen Li, Zhihua Chen, Songmin Ying, Huahao Shen

**Affiliations:** ^1^ Key Laboratory of Respiratory Disease of Zhejiang Province, Department of Respiratory and Critical Care Medicine, Second Affiliated Hospital of Zhejiang University School of Medicine, Hangzhou, China; ^2^ Department of Pharmacology, Zhejiang University School of Medicine, Hangzhou, China; ^3^ State Key Lab of Respiratory Disease, Guangzhou, China

**Keywords:** idiopathic pulmonary fibrosis, Bcl-2, ABT-199, macrophage, fibroblast

## Abstract

Idiopathic pulmonary fibrosis (IPF) is a distressing lung disorder with poor prognosis and high mortality rates. Limited therapeutic options for IPF is a major clinical challenge. Well-known for its anti-apoptotic properties, B-cell lymphoma 2 (Bcl-2) plays a critical role in the pathology of malignancies and inflammatory diseases, including IPF. In this study, we aimed to investigate the therapeutic effect of a Bcl-2 homology domain 3 mimetic inhibitor, ABT-199, on bleomycin (BLM)-induced pulmonary fibrosis in mice, and explore possible underlying mechanism. The lung inflammation and fibrosis model was established by intratracheal instillation of a single dose of BLM. We observed elevated Bcl-2 in the alveolar macrophages and fibroblasts derived from BLM-instilled mice from day 7. Further, we obtained *in vivo* evidence that early therapeutic treatment with Bcl-2 inhibitor ABT-199 from day 3, and late treatment from day 10, both alleviated airway inflammation and lung fibrosis induced by BLM. Our data suggest that ABT-199 might be an effective antifibrotic agent that interferes with profibrogenic cells, which may be a promising therapy in the treatment of clinical IPF patients.

## Introduction

IPF is an interstitial lung disease characterized pathologically by progressive scarring of lung tissue, increased fibroblast proliferation, excessive accumulation of extracellular matrix (ECM), exacerbation of lung inflammation, diffused destruction of alveolar structure and remodeling of the lung architecture (Todd et al., 2012). The clinical symptoms of IPF are progressive dyspnea, wheezing or panting which finally lead to respiratory failure (Chen et al., 2013). This disease is significantly correlated to a dismal outcome with a median survival time of 2–4 yr after diagnosis (Bjoraker et al., 1998). To date, there is no promising medication to effectively cure this disease, which makes it vital to find novel therapeutic targets to treat IPF.

Apoptosis plays a significant role in wound repair and pulmonary epithelial injury leading to fibrosis. Decreased apoptosis of inflammatory cells and fibroblasts results in chronic inflammation which injures the lung and promotes fibrogenesis (Polunovsky et al., 1993). Notably, a marked reduction in the apoptosis of alveolar macrophages plays an integral role in the pathogenesis of pulmonary fibrosis by triggering an immune response (Larson-Casey et al., 2016). There is evidence that the apoptosis rate of alveolar macrophages in bronchial alveolar lavage fluid (BALF) of IPF patients was decreased compared with normal subjects (Drakopanagiotakis et al., 2012). Further, alveolar macrophages could release important profibrotic mediators, such as transforming growth factor-β1 (TGF-β1) (Jain et al., 2013). Overexpression of active TGF-β1 would extend the survival time of lung fibroblasts, which is known to play a crucial role in the pathogenesis of fibrotic processes and ECM protein synthesis (Huang and Natarajan, 2015). Consequently, the persistent existence of lung fibroblasts induces prolonged and severe interstitial lung fibrosis (Sime et al., 1997; Zhou et al., 2013). Therefore, promoting apoptosis of alveolar macrophages and lung fibroblasts may be a potential strategy to decline proinflammatory cytokine production and accelerate the resolution of IPF.

Bcl-2 family proteins exist in the upstream of apoptotic pathways and have a pivotal role in the regulation of apoptosis. More than 20 members with diverse proapoptotic or anti-apoptotic functions are included in Bcl-2 family (Gross et al., 1999). Mounting evidence revealed that during pulmonary fibrosis, deregulation of Bcl-2 family proteins and imbalance between pro- and anti-apoptotic activities may determine the cellular susceptibility to apoptosis, among which Bcl-2 as an anti-apoptotic protein promotes cell survival. The expression of Bcl-2 differs in different cell types, for example, lower expression of Bcl-2 was observed in alveolar epithelial cell after intratracheally instillation of bleomycin (Oltvai et al., 1993). While in fibroblasts and macrophages, the expression of Bcl-2 increased (Kazufumi et al., 1997; Moodley et al., 2003; Nishimura et al., 2007). Thus, using the inhibitors against antiapoptotic Bcl-2 family proteins might prove a promising treatment option for IPF. One such example is the small molecule BH3 domain peptide mimetic ABT-199, a highly potent bioavailable Bcl-2 specific inhibitor (Oltersdorf et al., 2005). It was approved by the US Food and Drug Administration to treat chronic lymphocytic leukemia (Deeks, 2016). Studies have shown that ABT-199 has remarkable therapeutic effects on steroid-insensitive airway inflammation and particulate matter-induced lung inflammation (Tian et al., 2017; Geng et al., 2018). However, the therapeutic potential of ABT-199 against IPF has not been investigated till now. Hence, in the present study, we sought to explore whether Bcl-2 is a promising target for IPF and evaluate the antifibrotic effect of ABT-199 on BLM-induced pulmonary inflammation and lung fibrosis.

## Materials and Methods

### Reagents

ABT-199 was purchased from Selleckchem (TX, United States), and BLM was purchased from Hisun Pfizer Pharmaceuticals Co., Ltd (Zhejiang, China). Hydroxyproline (HYP) assay kit was purchased from Nanjing Jiancheng Biochemical Institute (Nanjing, China). Mouse TGF-β1 and interleukin-6 (IL-6) enzyme-linked immunosorbent assay (ELISA) kits used were from R&D (Minneapolis, MN, United States). Antibody against Bcl-2 (7382) was purchased from Santa Cruz Biotechnology (TX, United States). Antibodies against α-SMA (ab5694) and type I collagen (ab34710) were purchased from Abcam Inc. (Cambridge, MA, United States). BCA Protein Assay kit was purchased from Thermo Fisher Scientific (Waltham, MA, United States).

### Animal Model of BLM-induced Pulmonary Fibrosis

8-wk old, male C57BL/6 mice weighing 18–22 g were purchased from the Animal Center of Zhejiang University. The mice were housed in a Specific Pathogen Free (SPF) facility and provided with food and water ad libitum. The pulmonary fibrosis mouse model was established as previously described (Larson-Casey et al., 2016). Briefly, mice were anesthetized *via* intraperitoneal injection of 40 mg/kg pentobarbital sodium solution and then the skin and subcutaneous tissue overlying the proximal portion of the trachea were exposed by a 5 mm transversal incision to allow insertion into the trachea of a needle containing a single dose of BLM 50 μl (2.5 U/kg, BLM group) or the same volume of sterile saline (normal saline, NS group). These two groups were respectively divided into two subgroups, a control group, and ABT-199 treatment group. Mice in the latter group were treated through intratracheal administration of 2 μg/μl ABT-199, suspended in 50 μl PBS, while the mice in control group were treated with an equal volume of PBS. Mice were sacrificed at the indicated times after BLM/saline instillation and their lungs and BALF were collected. The animal study was reviewed and approved by the Institutional Animal Care and Use Committee of Zhejiang University. Experiments with animals were carried out in accordance with the guidelines of the Animal Care and Use Committee of the Zhejiang University School of Medicine.

### BALF Collection and Analysis

BALF was collected and centrifuged at 400 g for 10 min at 4°C. Supernatant was stored at −80°C for the analysis of cytokines, while cell pellets were resuspended in 200 μl PBS for total and differential cell counting, numbers of macrophages, lymphocytes and neutrophils in a total of 200 cells were counted and categorized under microscope, according to standard morphological criteria. Total BALF protein was measured using BCA Protein Assay Kit, according to the manufacturer’s instructions.

### Histological Examination

Lungs were fixed in 4% paraformaldehyde and embedded in paraffin. Sections were stained with Hematoxylin-Eosin (HE) or Masson’s trichrome for evaluation of fibrosis under a microscope. The Szapiel score was used to evaluate the degree of parenchymal alveolitis (Szapiel et al., 1979). The area analysis of fibrotic changes was performed according to Ashcroft scale (Ashcroft et al., 1988). Five randomly chosen fields within each lung section were observed at a magnification of ×100 or 200×. Two independent blind observers scored each specimen. A mean score of Szapiel or Ashcroft for each mouse was used for statistical analysis. For detection of fibroblasts and myofibroblasts, the slides were probed with antibodies against type I collagen (collagen I, 1:500) or α-smooth muscle actin (α-SMA, 1:600) as previously described (Rangarajan et al., 2018). Images were acquired on a fluorescence microscope and analyzed using ImageJ software.

### Hydroxyproline Assay

The collagen content of lungs was measured by HYP assay kits according to the manufacturer’s instructions. Briefly, 0.1 g of lung tissue was weighed and placed into a test tube, 3 ml of 6 mol/L HCl was added, and lungs were then digested for 12 h at 110°C. Later, the samples were neutralized by adjusting pH to 6.0–6.8 with NaOH. Subsequently, ddH_2_O was added to make a total volume of 10 ml. Absorbance was measured at 560 nm on a spectrophotometer and amount of HYP was calculated.

### Immunofluorescence

BAL cells were collected and fixed in 4% paraformaldehyde for 30 min, permeabilized in 0.2% Triton X-100 (LC262801; Sigma-Aldrich) for 5 min, and then blocked with 5% BSA (Sigma-Aldrich, B2064) for 1 h. Then cells were incubated with the antibody against Bcl-2 (1:1000) at 4°C overnight. Sections were stained with goat anti-mouse IgG secondary antibody (A28180; Invitrogen) (1:5000) for 30 min at room temperature. Slides were then washed and counterstained with DAPI. Finally, slides were observed under a fluorescence microscope (Olympus).

### Enzyme-linked Immunosorbent Assay

The levels of TGF-β1 and IL-6 in BALF and lung homogenate were measured by ELISA kits according to the manufacturer’s instructions.

### Real-time Quantitative PCR

Total RNA was extracted from lungs using Trizol reagent (Takara Biotechnology) according to the manufacturer’s instructions. RNA was then reverse transcribed into cDNA using Reverse Transcription Reagents (Takara Biotechnology). Real-time PCR was performed with the SYBR Green Master Mix (Takara Biotechnology) on a StepOne real-time PCR system (Applied Biosystems, Foster City, CA, United States). The relative expression of target genes was normalized against β-actin using the 2^−ΔΔCt^ method. Primer sequences used were as follows: *β-actin* (Forward: 5′-GTC​CAC​CGT​GTA​TGC​CTT​CT-3′, Reverse: 5′-CTC​CTG​GTG​TCC​GAA​CTG​AT-3′); *Il-6* (Forward: 5′-CTG​CAA​GAG​ACT​TCC​ATC​CAG-3′, Reverse: 5′-AGT​GGT​ATA​GAC​AGG​TCT​GTT​GG-3′); *Tgf-β1* (Forward: 5′-GCG​CTC​ACT​GCT​CTT​GTG​ACA-3′, Reverse: 5′-GCA​ATA​GTT​GGT​ATC​CAG​GGC​TCT-3′); *Tnf-α* (Forward: 5′-CAC​TTG​GTG​GTT​TGC​TAC​GA-3′, Reverse: 5′-GTG​GCC​CCT​GCC​ACA​AGC​AG-3′); *Il-1β* (Forward: 5′-GAT​CCA​CAC​TCT​CCA​GCT​GCA-3′, Reverse: 5′-CAA​CCA​ACA​AGT​GAT​ATT​CTC​CAT​G-3′).

### Statistical Analysis

Results are expressed as mean ± SD. All data were analyzed using GraphPad Prism Version 7.0 software. Statistical comparisons were performed using Student t-test for two-group comparison and one or two-way ANOVA with a Tukey’s *post hoc* test for multiple comparisons. *p* < 0.05 was considered to be statistically significant.

## Results

### The Expression of Bcl-2 Increased in BALF Cells and Fibroblasts in BLM-induced Pulmonary Fibrosis

To validate the inflammatory response of IPF *in vivo*, we first established a BLM-induced pulmonary inflammation mouse model by intratracheally instilling a single dose of BLM, BALF and lung tissues was collected on day 3, 7 and 14. The findings revealed that BLM induced lung inflammation was dominated by macrophages on day 7 (Supplementary Figure S1A). In addition, both the total number of BAL cells and the absolute number of differential cells in the BLM group increased significantly relative to the NS group (Supplementary Figure S1B).

**FIGURE 1:**
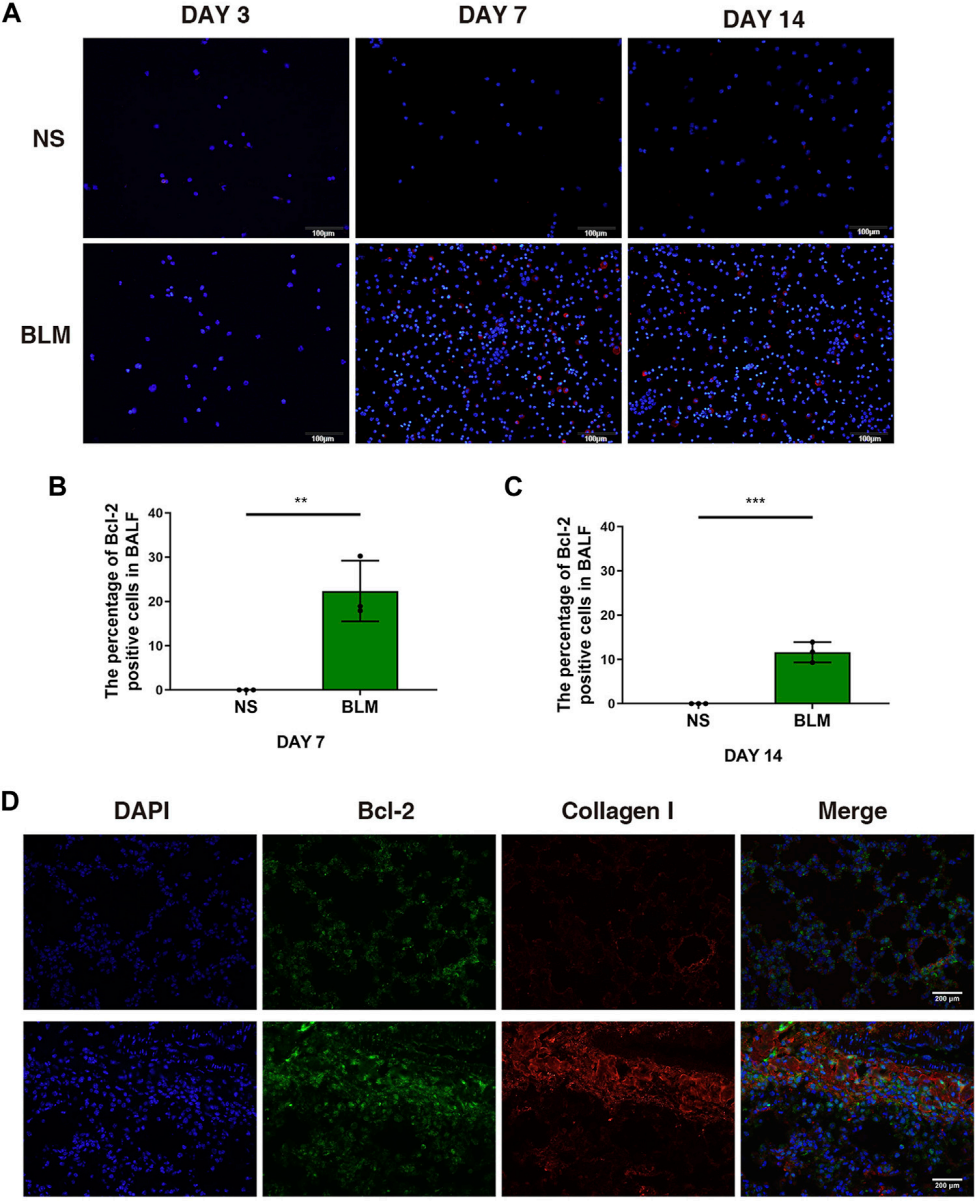
The expression of Bcl-2 was increased in BALF cells and fibroblasts in BLM-induced pulmonary fibrosis. **(A**–**C)** Intracellular Bcl-2 expression of BAL cells was assessed by immunofluorescence assay, and the percentage of Bcl-2 positive macrophages was determined. **(D)** Representative images of collagen I and Bcl-2 show fluorescent staining in lung tissues of mice induced by BLM at day 14. The data are presented as means ± SD of an experiment performed three times. **p < 0.01, ***p < 0.001. *n* = 5. BLM, bleomycin; BAL, bronchial alveolar lavage; NS, normal saline; Bcl-2, B-cell lymphoma-2.

We examined the mRNA expression level of Bcl2 in whole lung tissue by qPCR, the results showed no significant statistical difference after BLM administration (Supplementary Figures S2A–C). Of note, the percentage of Bcl-2 positive macrophages examined by immunofluorescence increased after BLM instillation on day 7 and 14 (Figures 1A–C), suggesting that macrophages accumulate in BALF during inflammatory phase with the expression of Bcl-2 increasing significantly. Moreover, fibroblasts began to grow after the instillation of BLM for 7–14 days, we observed that the expression of Bcl-2 in fibroblasts increased on day 14 (Figure 1D).

### Early ABT-199 Treatment Attenuated Lung Inflammation and in the Early Inflammation Phase

**FIGURE 2:**
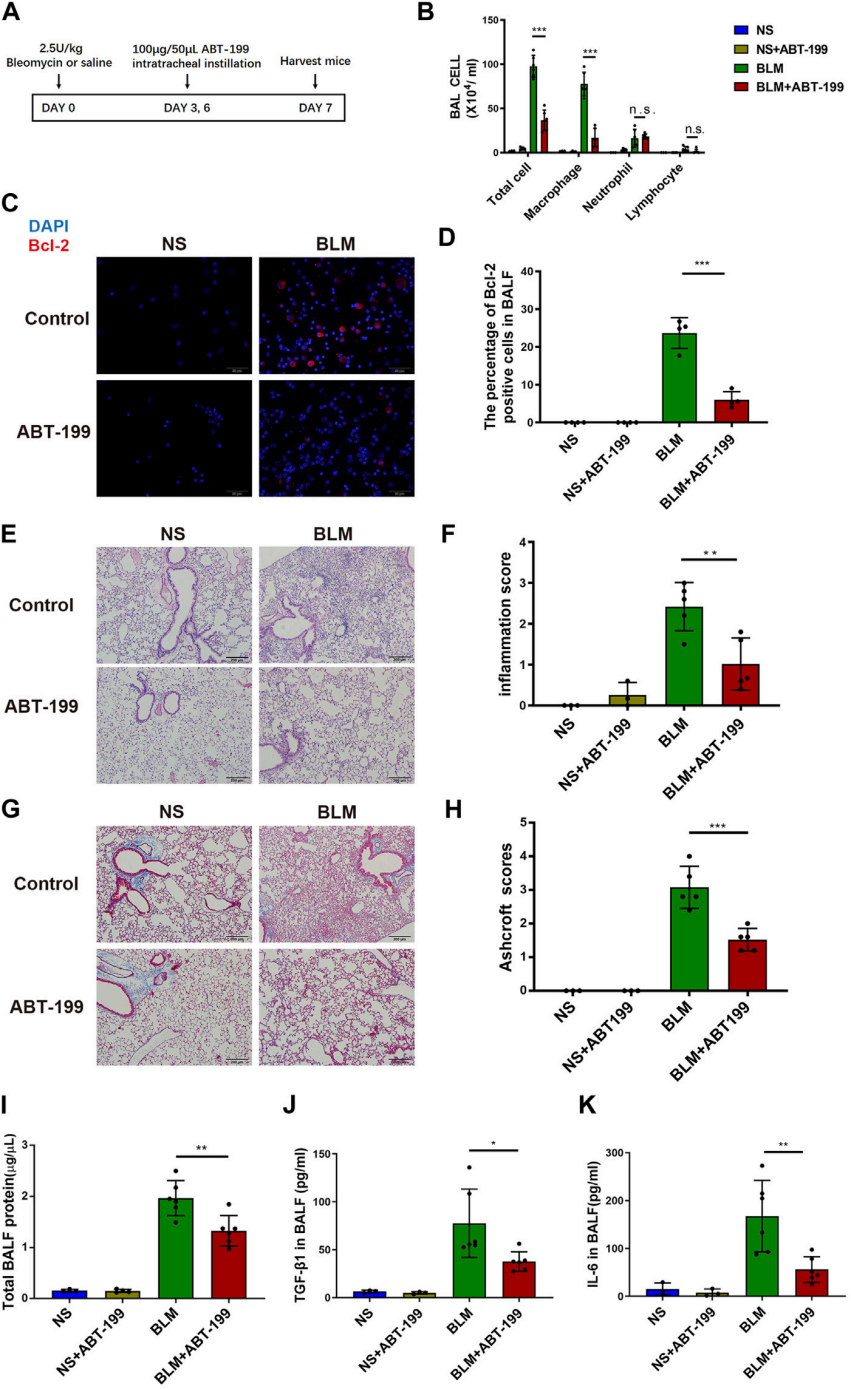
Early ABT-199 treatment attenuated BLM-induced lung inflammation at day 7. **(A)** Treatment scheme. **(B)** After 7 days, the total number of inflammatory cells was quantified and the number of differential cells was calculated. **(C)** Intracellular Bcl-2 expression of BAL cells was assessed by immunofluorescence assay on day 7. **(D)** The percentage of Bcl-2 positive macrophages was determined. **(E)** Representative images of lung sections stained with hematoxylin and eosin (H&E). **(F)** Degree of parenchymal alveolitis was evaluated using Szapiel score. **(G)** Representative images of lung sections stained with Masson’s trichrome. **(H)** The area analysis of fibrotic changes according to Ashcroft scale. **(I)** Total BALF protein on day 7 was measured using BCA Protein Assay. **(J**,**K)** Expressions of TGF-β1 and IL-6 levels in BALF on day 7 were determined using ELISA. The data are presented as means ± SD of an experiment performed three times. n.s., not significant, *p < 0.05, **p < 0.01, ***p < 0.001. *n* = 3–6. BLM, bleomycin; BAL, bronchial alveolar lavage; NS, normal saline; Bcl-2, B-cell lymphoma-2.

To test if targeting Bcl-2 could alleviate the inflammatory response after BLM injury, we applied the Bcl-2 inhibitor ABT-199 *in vivo*. ABT-199 (100 μg/mouse) was administrated to mice on day 2 after BLM instillation, and mice were sacrificed on day 3 (Supplementary Figure S3A), the results showed no obvious inflammation induced by BLM and no significant effect following ABT-199 administration (Supplementary Figures S3B–F). Next, ABT-199 was then administrated to mice every 3 days from day 3 after BLM instillation, and mice were sacrificed on days 7 (Figure 2A). Inflammatory cell recruitment and release of cytokines were studied to determine the effects of ABT-199 in BLM-induced lung inflammation. As expected, the number of total cells macrophages in the BALF of ABT-199 treated mice decreased significantly compared to the BLM-only treated group (Figure 2B).

In addition, the percentage of Bcl-2 positive macrophages in BALF decreased after 7 days of treatment with ABT-199 (Figures 2C,D). The attenuation of inflammation in ABT-199-treated mice was further supported by histological analysis by H&E staining (Figures 2E,F), which demonstrated less inflammation and reduced alveolar wall thickness at indicated time points. Results of Masson’s trichrome staining revealed that the ABT-199-treated fibrotic mice had a marked reduction in collagen accumulation and preserved lung architecture as shown by the lower Ashcroft scores (Figures 2G,H). To further explore the effect of ABT-199 therapy, we then assessed the level of inflammatory response. Administration of ABT-199 also reduced the BALF protein content in BLM-induced lung injury (Figure 2I). Moreover, ABT-199 treatment significantly reduced the concentration of TGF-β1 and IL-6 protein in BALF on day 7 (Figures 2J,K).

**FIGURE 3:**
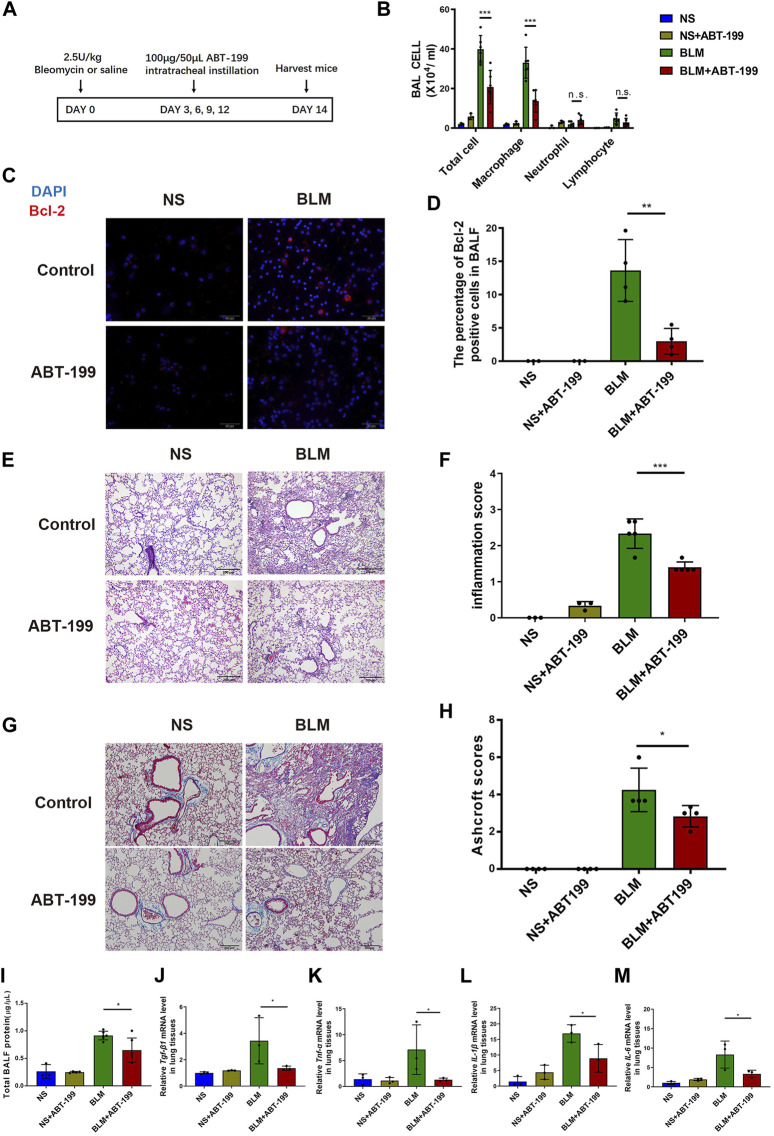
Early ABT-199 treatment attenuated BLM-induced lung inflammation at day 14. **(A)** Treatment scheme. **(B)** After 14 days, the total number of inflammatory cells was quantified and the number of differential cells was calculated. **(C)** Intracellular Bcl-2 expression of BAL cells was assessed by immunofluorescence assay on day 14. **(D)** The percentage of Bcl-2 positive macrophages was determined. **(E)** Representative images of lung sections stained with hematoxylin and eosin (H&E). **(F)** Degree of parenchymal alveolitis was evaluated using Szapiel score. **(G)** Representative images of lung sections stained with Masson’s trichrome. **(H)** The area analysis of fibrotic changes according to Ashcroft scale. **(I)** Total BALF protein on day 14 was measured using BCA Protein Assay. **(J**–**M)** The relative levels of *Tgf-β1, Tnf-α, Il-1β, and Il-6* mRNA transcripts were determined using quantitative PCR. The data are presented as means ± SD of an experiment performed three times. n.s., not significant, *p < 0.05, **p < 0.01, ***p < 0.001. *n* = 3–6. BLM, bleomycin; BAL, bronchial alveolar lavage; NS, normal saline; TGF-β1, transforming growth factor-β1; TNF-α, tumor necrosis factor-α; IL-1β, interleukin-1β; IL-6, interleukin-6.

The continuous attenuation of inflammation in ABT-199-treated mice was further confirmed on day 14 (Figure 3A). Less infiltration of inflammatory cells, in which reduced Bcl2 positive macrophage was observed in BALF (Figures 3B–D). The inflammation score and Ashcroft score for mice which received ABT-199 was much lower compared to that for BLM-only treated mice (Figures 3E–H), accompanied by reduced BALF protein and alleviation of fibrosis factor expression (Figures 3I–M). *In vitro*, we also treated the mouse peritoneal macrophages with ABT-199 and found that 10 μM ABT-199 could induce the apoptosis (Supplementary Figures S4A,B). Conclusively, these data indicate that ABT-199 is effective against BLM-induced lung inflammatory response by reducing the number of BAL macrophages.

### ABT-199 Significantly Decreased Bcl-2 and Attenuates BLM-induced Lung Fibrosis in Mice

**FIGURE 4:**
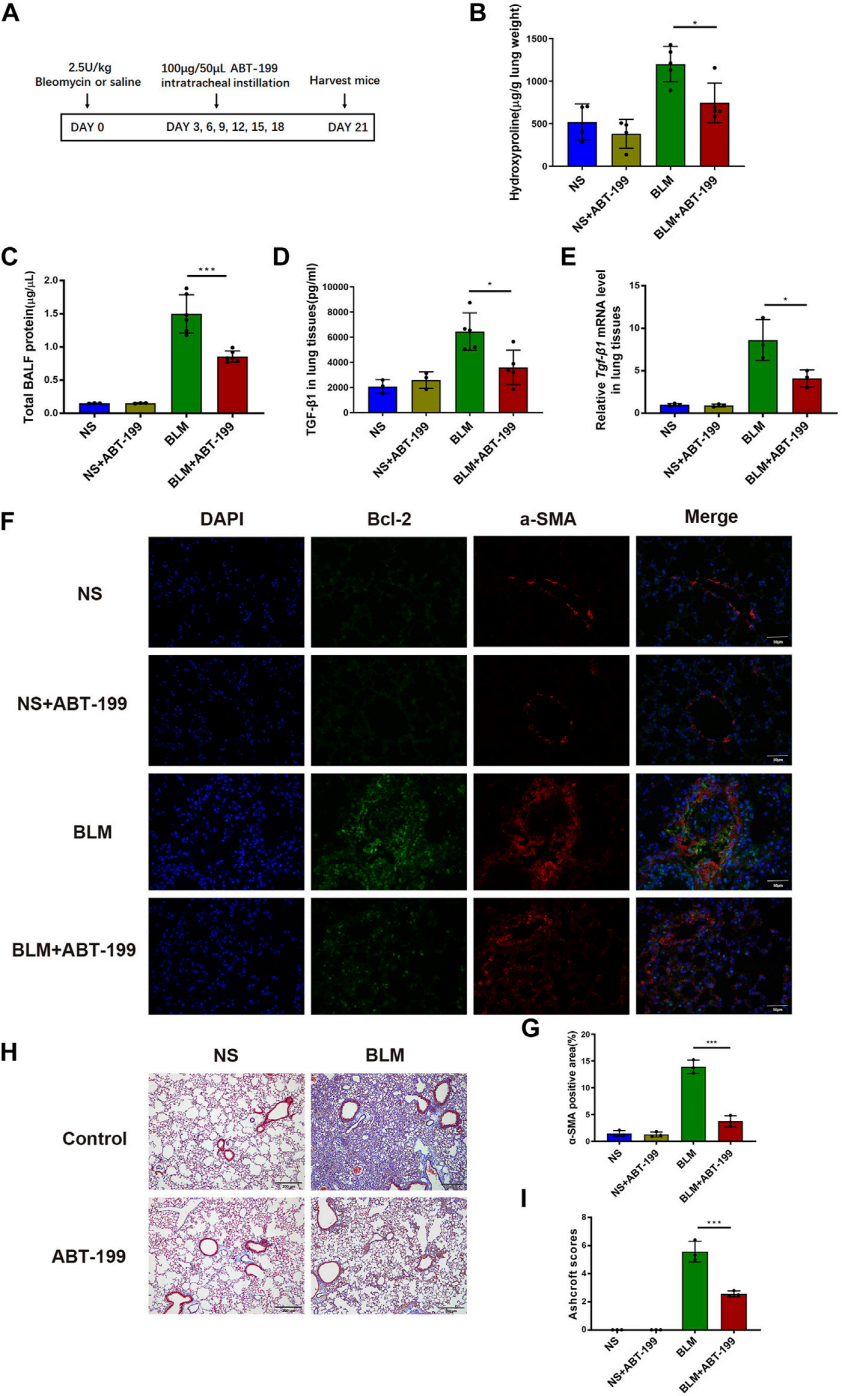
ABT-199 alleviates BLM-induced lung fibrosis. **(A)** Treatment scheme. **(B)** Hydroxyproline contents in lung tissues. **(C)** Total BALF protein on day 21 was measured using BCA Protein Assay. **(D**,**E)** Expression of TGF-β1 level in the lung tissue were determined using ELISA and real-time PCR. **(F)** Representative images of myofibroblasts in lung tissues by immunofluorescence staining of α-SMA and Bcl-2. **(G)** Quantitative analysis of α-SMA positive areas in lung sections. **(H)** Representative images of lung sections stained with Masson’s trichrome. **(I)** The area analysis of fibrotic changes according to Ashcroft scale. The data are presented as means ± SD of an experiment performed three times. *p < 0.05, ***p < 0.001. *n* = 3–6. BLM, bleomycin; BAL, bronchial alveolar lavage; NS, normal saline; TGF-β1, transforming growth factor-β1; α-SMA, alpha smooth muscle actin.

To evaluate the antifibrotic effects of ABT-199 against BLM-induced pulmonary fibrosis in mice, we established a 21 days BLM-induced pulmonary fibrosis mouse model and intratracheally instilled ABT-199 (100 μg/mouse) every 3 days, starting from the third day after BLM administration (Figure 4A). The lung hydroxyproline content and BALF protein concentration of fibrotic mice were significantly reduced by ABT-199 treatment (Figures 4B,C). Analysis of lung extracts revealed decreased mRNA and protein levels of TGF-β1 in mice following treatment with ABT-199 (Figures 4D,E). Furthermore, Bcl2 expression, increased in BLM group and reduced following ABT-199 administration, was shown to be co-localized with α-SMA (Figure 4F). Also, quantitative analysis showed that the α-SMA positive areas were smaller in the lungs of ABT-199-treated fibrotic mice than that of fibrotic mice (Figure 4G), suggesting a reduction in myofibroblasts. The results of Masson’s trichrome staining revealed that the ABT-199-treated fibrotic mice had a marked reduction in collagen accumulation and preserved lung architecture as shown by the lower Ashcroft scores (Figures 4H,I), which were consistent with the above findings. Collectively, these results confirmed the therapeutic effect of ABT-199 against BLM-induced pulmonary fibrosis in mice.

### ABT-199 Attenuated Established Lung Fibrosis in BLM-injured Mice

**FIGURE 5:**
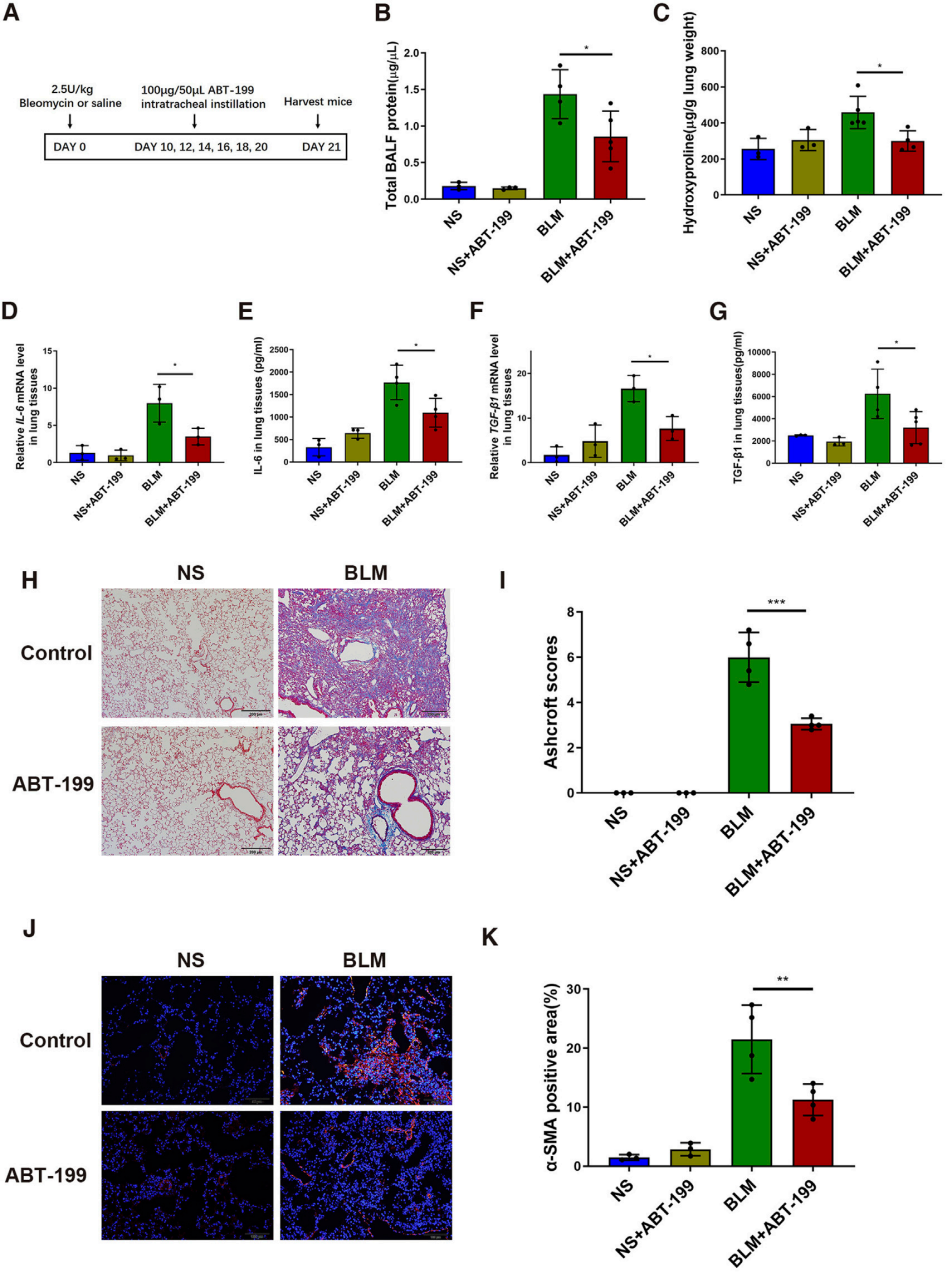
ABT-199 reverses the established lung fibrosis in BLM-injured mice. **(A)** Treatment scheme. **(B)** Total BALF protein was measured using BCA Protein Assay. **(C)** Hydroxyproline contents in lung tissues. **(D**,**E)** Expression level of IL-6 in the lung tissue was determined using real-time PCR and ELISA. **(F**,**G)** Expression level of TGF-β1 in the lung tissue was determined using real-time PCR and ELISA. **(H)** Representative images of lung sections stained with Masson’s trichrome. **(I)** The area analysis of fibrotic changes according to Ashcroft scale. **(J)** Representative images of myofibroblasts in lung tissues by immunofluorescence staining of α-SMA. **(K)** Quantitative analysis of α-SMA positive areas in lung sections. The data are presented as means ± SD of an experiment performed three times. *p < 0.05, **p < 0.01, ***p < 0.001. *n* = 3–6. BLM, bleomycin; Bcl-2, B-cell lymphoma-2; BAL, bronchial alveolar lavage; NS, normal saline; TGF-β1, transforming growth factor-β1; IL-6, interleukin-6.

To find out whether the inhibition of Bcl-2 protein could attenuate established lung fibrosis during the post-inflammatory phase, we administered ABT-199 every 2 days from day 10 (Figure 5A). ABT-199 administration resulted in decreased number of infiltrating macrophages (Supplementary Figure S5). Notably, even after collagen has already been deposited, ABT-199 significantly reduced the over-production of total BALF protein the over-accumulation of HYP (Figures 5B,C) induced by BLM. Meanwhile, the results of qPCR and ELISA showed that ABT-199 significantly downregulated the expression of pro-fibrotic cytokines (IL-6 and TGF-β1) (Figures 5D–G). Histological analysis of lung by Masson’s trichrome staining revealed that ABT-199 could effectively attenuate BLM-induced pulmonary fibrosis, mainly by reducing the thickness of alveolar walls and collagen deposition (Figures 5H,I). Reduction in myofibroblasts was also shown by the α-SMA positive areas that were smaller in the lungs of ABT-199-treated fibrotic mice than that of fibrotic mice (Figures 5J,K). *In vitro*, we also treated mouse embryonic fibroblasts with ABT-199 and found that 100 μM ABT-199 could induce the apoptosis (Supplementary Figures S6A,B). In summary, our data confirm the antifibrotic effect of ABT-199 on established lung fibrosis.

## Discussion

At present, the medications used clinically to treat IPF are insufficient to prevent or reverse the progression of the disease. Therefore, the development of novel therapeutic approaches against IPF is mandatory. Bcl-2, which is known as an inhibitor of apoptosis promotes cell survival (Llambi and Green, 2011). There is a growing body of evidence that suggests a crucial role for Bcl-2 in the pathogenesis of inflammation, apoptosis and fibrosis induced by various factors in the interstitial lung diseases. In this study, we demonstrated that in a mouse model of BLM-induced pulmonary fibrosis, numerous macrophages were recruited to BALF and fibroblasts proliferated in the lungs. Higher expressions of Bcl-2 was detected in alveolar macrophages and lung fibroblasts, which displays similar expression pattern with α-SMA and the severity of lung fibrosis. Furthermore, we found ABT-199 mediated selective inhibition of Bcl-2 significantly attenuated lung inflammation and fibrosis, even with a late treatment from day 10, when the fibrosis had already been established.

Different experimental models have been developed for understanding the role of Bcl-2 protein in the regulation of apoptosis through fibrosis development, including bleomycin, asbestos, silica, paraquate, chemokine and radiation, among which, the bleomycin model of pulmonary fibrosis is the best characterized murine model. The pathogenesis of IPF is divided into two phases, named the early inflammatory response and the late fibrogenesis. The inflammation phase which is mediated by various inflammatory cells is the initial response after BLM stimulation. In this study, a significant increase in inflammatory cells in BALF from BLM treated mice on day 7 and 14 were detected, especially macrophages, which are known to be the primary source of a variety of pro-inflammatory and fibrogenic cytokines and growth factors (Kendall and Feghali-Bostwick, 2014; Li et al., 2019). Previous studies showed that specific macrophage depletion attenuated lung injury *in vivo* (Redente et al., 2014). Therefore, whether macrophage apoptosis induction be a potential anti-fibrosis therapy remains to be critical and rarely studied.

Our results revealed that the Bcl-2 in alveolar macrophages was increased significantly, suggesting accumulation of macrophages due to their apoptosis resistance in the airway during inflammatory phase. Thus, we wondered if the treatment with Bcl-2 inhibitor ABT-199 would decrease inflammation response in a model of BLM-induced pulmonary fibrosis. Our results confirm that ABT-199 reduced the number of BAL macrophages in the early inflammation phase of lung fibrosis, which might due to direct pro-apoptotic effect on macrophages. In addition, we also observed a decline in the level of various harmful growth factors and cytokines, which is consistent with the fact that macrophages are known to initiate an inflammatory response after injury by secreting these factors, which contribute to the further amplification of inflammatory processes (Bitterman et al., 1982; Lemaire et al., 1986).

Although suppressing inflammatory response has been regarded as an important clinical strategy to treat IPF (Hara et al., 2012). More and more researches proved that anti-inflammatory treatments may not be effective for all IPF patients. The inflammation is significant in early phase of IPF but relieved in late fibrosis phase, which may lead to the failure of traditional anti-inflammatory therapy. Thus, the anti-fibrosis effect of ABT-199 may not be entirely dependent on the inhibition of inflammatory response. Our observation suggested that the lung fibroblasts were established on day 14 and showed increased apoptosis resistance, with high expression of Bcl-2, indicates that fibroblasts were also a target of ABT-199. Therefore, we aimed to evaluate whether ABT-199 could reverse the already existing fibrosis using a late-ABT199 treatment model. As active fibrosis phase is between 7 and 14 days, significantly increased collagen deposition is observed on day 14 (Peng et al., 2013). For this reason, we intratracheally instilled ABT-199 from 10 days after BLM administration, when lung fibrosis was already established. Our data showed that BLM-induced fibrosis was significantly attenuated. Therefore, a direct effect of ABT-199 on fibroblast could have accounted for the antifibrotic effects observed.

Finally, we originally illustrate the potential therapeutic effect of ABT-199 against BLM-induced pulmonary fibrosis in mice, and emphasized that both early and late treatment with ABT-199 attenuated pulmonary fibrosis through its anti-inflammatory, along with anticoagulant effects. Although we propose that ABT-199 could be used as a new medicinal approach against fibrotic diseases such as IPF, further studies are still required to reveal its more comprehensive effects and mechanism of action.

## Data Availability

The original contributions presented in the study are included in the article/Supplementary Material, further inquiries can be directed to the corresponding authors.

## Abbreviations

BALF, bronchial alveolar lavage fluid; Bcl-2, B-cell lymphoma 2; BLM, bleomycin; Collagen I, type I collagen; ECM, extracellular matrix; ELISA, enzyme-linked immunosorbent assay; HE, Hematoxylin-Eosin; HYP, hydroxyproline; IL-1β, interleukin-1β; IPF, Idiopathic pulmonary fibrosis; NS, normal saline; α-SMA, α-smooth muscle actin; SPF, Specific Pathogen Free; TGF-β1, transforming growth factor-β1; TNF-α, tumor necrosis factor-α.
