# Supplementary material for: Therapeutic Effects of the Bcl-2 Inhibitor on Bleomycin-induced Pulmonary Fibrosis in Mice
Source: Front Mol Biosci. 2021 Oct 7;8:645846. doi: 10.3389/fmolb.2021.645846 (PMC8529052; doi:10.3389/fmolb.2021.645846)
Supplement: Supplementary file 1 [file DataSheet1.docx]

**Supplementary Information**

**
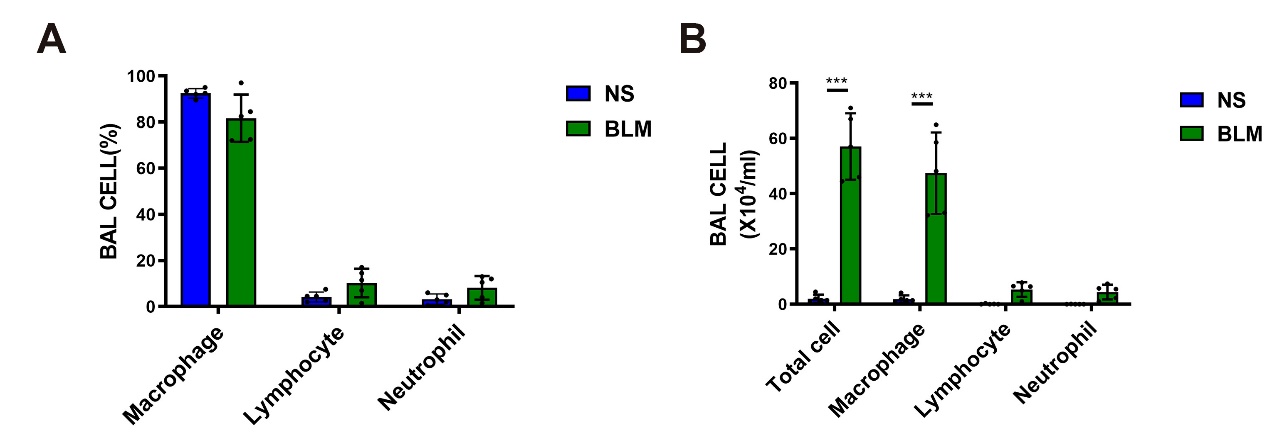
**

**Supplementary Figure 1.** **BLM-induced lung inflammation was dominated by macrophages. (A)** Differential cell percentages in BALF on day 7. **(B)** The total number of BAL cells and the absolute number of differential cells in BALF on day 7. The data are presented as means ± SD. ***p<0.001. Representative results from three independent experiments are shown.


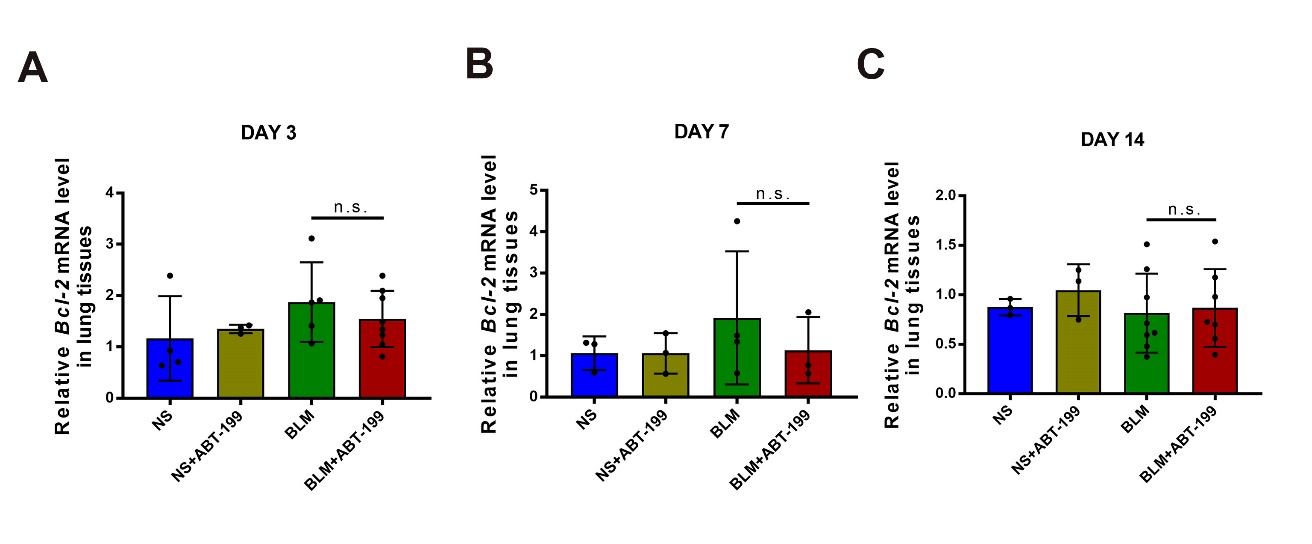


**Supplementary Figure 2. The effect of ABT-199 on Bcl-2 mRNA expression at different time points. (A)** The relative level of *Bcl-2* mRNA transcripts on day 3 was determined using quantitative PCR. **(B)** The relative level of *Bcl-2* mRNA transcripts on day 7 was determined using quantitative PCR. **(C)** The relative level of *Bcl-2* mRNA transcripts on day 14 was determined using quantitative PCR. The data are presented as means ± SD. n.s., not significant. Representative results from three independent experiments are shown.


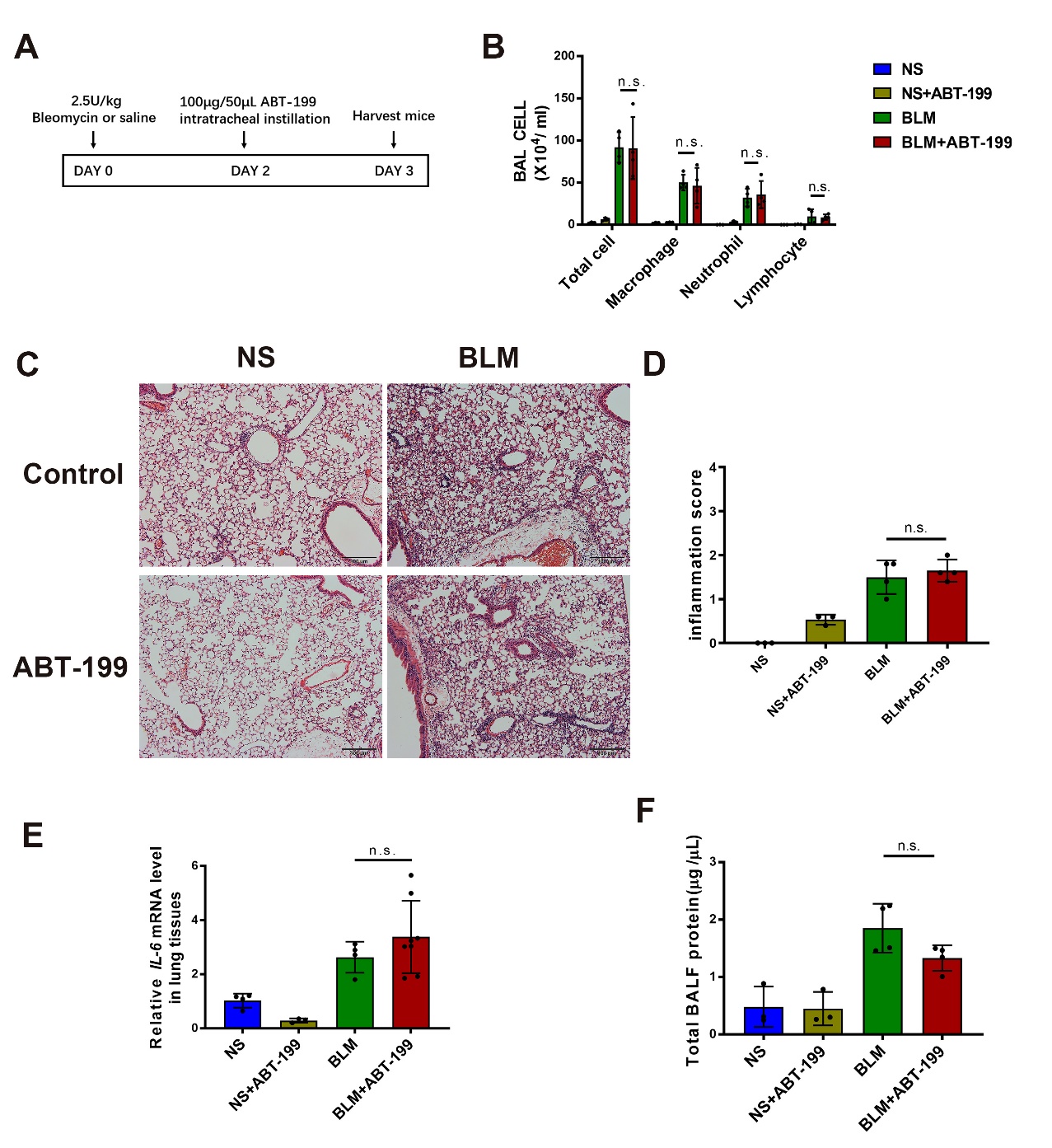


**Supplementary Figure 3. The effect of ABT-199 on BLM-induced lung inflammation on day 3.** **(A)** Treatment scheme. **(B)** The total number of BAL cells and the absolute number of differential cells in BALF on day 3. **(C)** Representative images of lung sections stained with hematoxylin and eosin (H&E). **(D)** Degree of parenchymal alveolitis was evaluated using Szapiel score. **(E)**The relative level of *Il-6* mRNA transcripts was determined using quantitative PCR. **(F)** BAL collagen on day 3 was measured using BCA Protein Assay. The data are presented as means ± SD. n.s., not significant. Representative results from three independent experiments are shown.


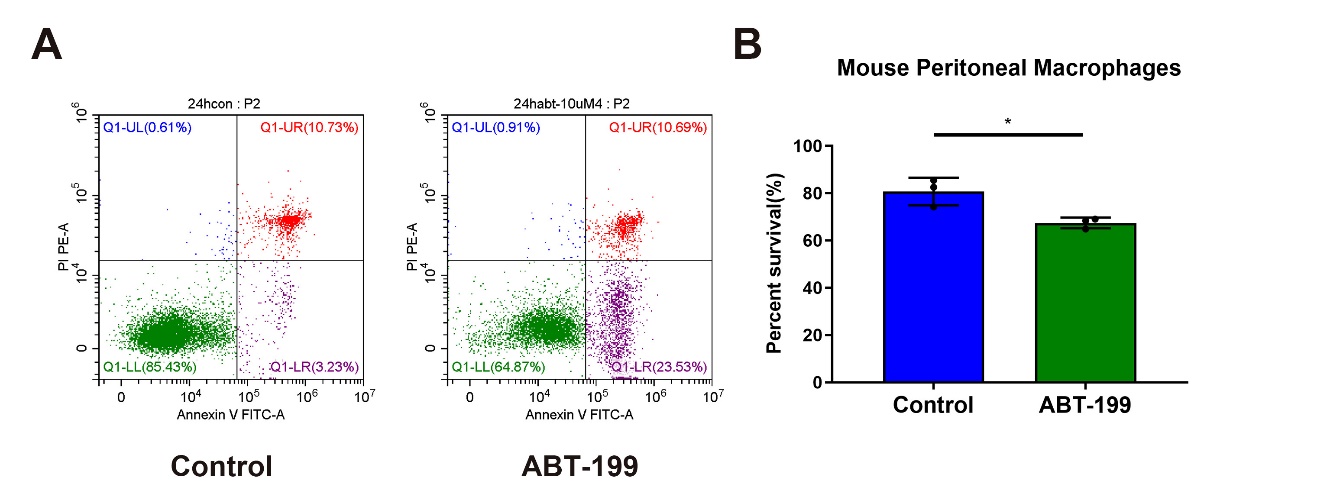


**Supplementary Figure 4.** **ABT-199 induces apoptosis of macrophages *in vitro*. (A-B)** Apoptosis level of mouse peritoneal macrophages induced by ABT-199 (10μM) was determined with Annexin V and PI staining by flow cytometry. The data are presented as means ± SD. *p<0.05. Representative results from three independent experiments are shown.


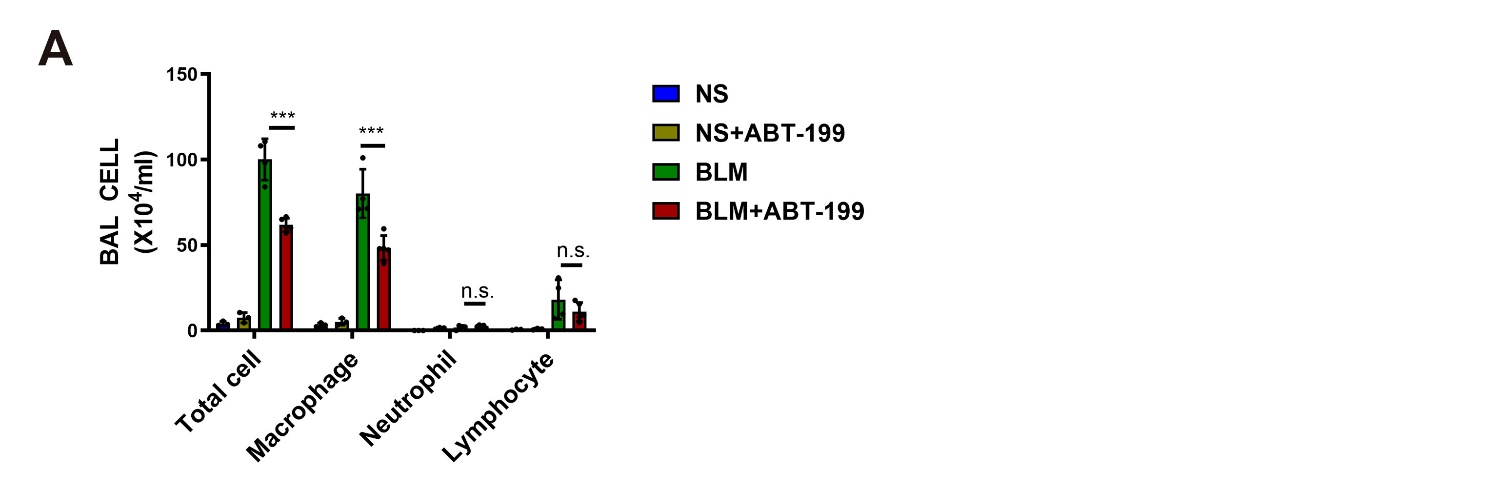


**Supplementary Figure 5. Cell differential analysis in BALF cells with late ABT-199 treatment. (A)** After 21 days, the total number of inflammatory cells was quantified and the number of differential cells was calculated. The data are presented as means ± SD. ***p<0.001. Representative results from three independent experiments are shown.

**
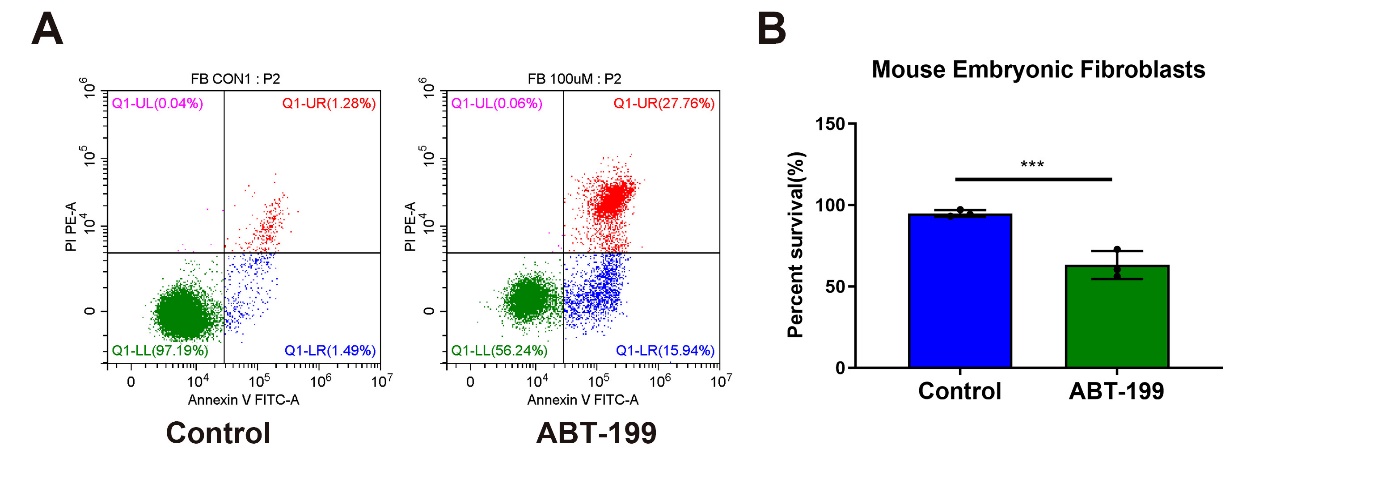
**

**Supplementary Figure 6.** **ABT-199 induces apoptosis of fibroblasts *in vitro*. (A-B)** Apoptosis level of mouse embryonic fibroblasts induced by ABT-199 (100μM) was determined with Annexin V and PI staining by flow cytometry. The data are presented as means ± SD. ***p<0.001. Representative results from three independent experiments are shown.
